# Sustainable intensification of agriculture for human prosperity and global sustainability

**DOI:** 10.1007/s13280-016-0793-6

**Published:** 2016-07-12

**Authors:** Johan Rockström, John Williams, Gretchen Daily, Andrew Noble, Nathanial Matthews, Line Gordon, Hanna Wetterstrand, Fabrice DeClerck, Mihir Shah, Pasquale Steduto, Charlotte de Fraiture, Nuhu Hatibu, Olcay Unver, Jeremy Bird, Lindiwe Sibanda, Jimmy Smith

**Affiliations:** 1 Stockholm Resilience Centre, Stockholm University, Stockholm, Sweden; 2 Crawford School of Public Policy, Australian National University, Canberra, Australia; 3 Department of Biology, Stanford University, Stanford, USA; 4 ICARDA, Amman, Jordan; 5 CGIAR Research Program on Water, Land and Ecosystems, Battaramulla, Sri Lanka; 6 Bioversity International, Montpellier, France; 7 Bharat Rural Livelihoods Foundation, New Delhi, India; 8 FAO, Land and Water Division, Rome, Italy; 9 Unesco-IHE, Delft, Netherlands; 10 Kilimo Trust, Kampala, Uganda; 11 IWMI, Battaramulla, Sri Lanka; 12 FANRPAN, Pretoria, South Africa; 13 ILRI, Nairobi, Kenya

**Keywords:** Agriculture development, Anthropocene, Global sustainability, Livelihoods, Resilience, Sustainable intensification

## Abstract

There is an ongoing debate on what constitutes sustainable intensification of agriculture (*SIA*). In this paper, we propose that a paradigm for sustainable intensification can be defined and translated into an operational framework for agricultural development. We argue that this paradigm must now be defined—at all scales—in the context of rapidly rising global environmental changes in the Anthropocene, while focusing on eradicating poverty and hunger and contributing to human wellbeing. The criteria and approach we propose, for a paradigm shift towards sustainable intensification of agriculture, integrates the dual and interdependent goals of using sustainable practices to meet rising human needs while contributing to resilience and sustainability of landscapes, the biosphere, and the Earth system. Both of these, in turn, are required to sustain the future viability of agriculture. This paradigm shift aims at repositioning world agriculture from its current role as the world’s single largest driver of global environmental change, to becoming a key contributor of a global transition to a sustainable world within a safe operating space on Earth.

## Introduction

A global food revolution based on a new paradigm for agricultural development is urgently required. Without this shift, we are unlikely to attain the twin objectives of feeding humanity and living within boundaries of biophysical processes that define the safe operating space of a stable and resilient Earth system (Steffen et al. 2015b). Global sustainability is increasingly understood as a prerequisite to attain human development (UN GSP 2012) at all scales, from local farming communities to cities, nations, and the world (Folke et al. 2005). The reason is that we have entered a new geological epoch, the Anthropocene, where human pressures are causing rising global environmental risks and for the first time constitute the largest driver of planetary change (Steffen et al. 2007, 2015b). Agriculture is at the heart of this challenge. It is the world’s single largest driver of global environmental change (Tilman et al. 2001; Foley et al. 2005; Godfray and Garnett 2014; Kuyper and Struik 2014) and, at the same time, is most affected by these changes (IPCC 2014). Agriculture is the key to attaining the UN Sustainable Development Goals of eradicating hunger and securing food for a growing world population of 9–10 billion by 2050, which may require an increase in global food production of between 60 and 110 % (Foley et al. 2005; IAASTD 2008; Tilman et al. 2011; Pardey et al. 2014) in a world of rising global environmental risks. Agriculture is also the direct livelihood of 2.5 billion smallholder farmers (FAO 2013a), and the resilience of these livelihoods to rising shocks and stresses is currently gravely under-addressed (FAO 2013b).

Together, these insights provide a strong scientific justification for a shift from our current paradigm for agriculture of focusing on productivity first and sustainability as a question of reducing environmental impacts, to a paradigm where sustainability constitutes the core strategy for agricultural development. The planetary boundary definition of a safe operating space for a stable and resilient Earth system provides an operational framework for defining what constitutes sustainable agriculture. It has been proposed (Keating et al. 2014) that the “safe operating space” exploration of food security (Beddington et al. 2012), based on these principles (Rockström et al. 2009), analytically frames the problem and describes the interconnected forces of population growth, consumption growth, environmental change, and food security.

The definition of a biophysical safe operating space of the Earth system, within which it has a high likelihood of remaining in a stable inter-glacial state, emerges from the advancements in Earth system science over the past decades, providing evidence of interactions, feedbacks, and thresholds among environmental processes that regulate the Earth system (Lenton et al. 2008), and the conclusion that humanity has entered a new geological Epoch, the Anthropocene (Waters et al. 2016), where the world constitutes the largest driver of change on Earth.

Therefore, in the Anthropocene, humanity faces the imperative question of how to transform agriculture that feeds the world, contributes to eradicate poverty, and contributes to a stable planet. Given the decisive role of world agriculture on human development and on Earth system processes, we argue in this paper that sustainable agriculture is the only strategy that can deliver productivity enhancements to meet rising food needs and enable an Earth system operating within planetary boundaries.

There is a well-documented debate (Garnett and Godfray 2012; Kuyper and Struik 2014) on what constitutes sustainable intensification of agriculture (*SIA*) (Garnett et al. 2013; Godfray and Garnett 2014; Struik et al. 2014), its evolution (Kuyper and Struik 2014; Struik et al. 2014), and its role in addressing global food security (van Noordwijk and Brussaard 2014).

Here, *SIA* is largely on how to enhance agricultural productivity while reducing its environmental impacts (Conway 1997; Godfray and Garnett 2014; Kuyper and Struik 2014). The task is how to produce more food with fewer resources. Sustainable intensification, in this context, seeks to increase agricultural output while keeping the ecological footprint as small as possible. This is, in no doubt, a useful and relatively important feature of sustainable agriculture, particularly as mainstream agriculture development still concentrates on productivity and places limited focus on sustainability. It remains focused though on avenues for resource efficiency, e.g., based on assumptions that efficiency in water and fertilizer use represents the avenues towards sustainable agriculture. Particularly in agricultural development in poverty-stricken regions, this “productivity first” paradigm, while potentially reducing environmental impacts, prevails.

There is an urgent need to shift this around and instead use sustainable principles as the entry point for generating productivity enhancements, which fundamentally requires real progress in increasing agricultural output by capitalizing on ecological processes in agro-ecosystems (Struik et al. 2014; van Noordwijk and Brussaard 2014). This can be achieved by managing farmers’ fields, watersheds, landscapes, and regions using strategies and practices that maintain biophysical stability and uphold critical feedbacks, such as moisture feedback from forests generating downwind rainfall (Gordon et al. 2008) and carbon sinks in soils and biomass (Le Quéré et al. 2015).

Incorporating ecological landscape approaches that make smart use of the natural functionalities that ecosystems offer is now an important part of the development of sustainable intensification of agriculture. The aim is to design multi-functional agro-ecosystems that are both sustained *by* nature and sustainable *in* their nature (Tittonell 2014).

In this paper, we propose that a new paradigm for *SIA* can be quantitatively defined from scientific advancements of the Anthropocene and biosphere resilience and translated into an operational framework for agricultural development. At its foundation, the new paradigm recognizes that the biophysical boundaries of Planet Earth impose a hierarchy of criteria on the definition of sustainability: sustainability is not a relative concept or an act of balancing competing claims; it sets absolute biophysical limits. It is only within such biophysically defined boundaries, such as operating within a 1.5 °C global carbon budget or within environmental water flows for river basins, which—as far as our current scientific knowledge shows—we stand a high probability of avoiding irreversible shifts in environmental conditions. The planetary boundary analysis sets the boundary for a stable climate system at 350 ppm of CO_2_ (uncertainty range of 350–450 ppm) or maximum 1 W/m^2^ of climate forcing (uncertainty range 1–1.5 W/m^2^), which translates to an average global temperature rise of approximately 1.5 °C (Rogelj et al. 2015; Steffen et al. 2015a). As has been suggested, only by defining development within such technically defined criteria or boundaries, social and economic trade-offs can be assessed (Fischer et al. 2007). Recent works (Jackson et al. 2012; Tittonell 2014; van Noordwijk and Brussaard 2014) signal mechanisms and demonstrate principles that suggest that such a transformative approach to *SIA* is possible and this paper presents examples of ways forward.

We suggest adding a new dimension to sustainable agricultural development, namely managing natural capital for long-term productivity and social–ecological resilience at field, watershed, and regional scales, in agricultural systems that operate within planetary boundaries to safeguard Earth system.

Our approach builds on existing research and the current evolution of the frameworks for *SIA* giving further emphasis to land-use planning and management of natural capital in both agro-ecosystems and natural ecosystems across scales. A resilience (capacity to deal with shocks and stress) and Earth system (in the Anthropocene) focus is key to deal with a rising frequency of multiple shocks triggered by regional and global changes unprecedented in human history.

Furthermore, such a comprehensive sustainability paradigm, which not only minimizes environmental impacts but also uses sustainability as the strategy to raise productivity, improve livelihoods, and build resilience and Earth system stability, must meet the dramatic rise in food requirements from a world population of nearly 10 billion by 2050, which most likely will reach 11 billion by the end of the century (Gerland et al. 2014). Together, these challenges—the social dimension of meeting rapidly rising food requirements and the ecological dimension of building agricultural resilience and Earth system stability—form a social–ecological framework for sustainable intensification of world agriculture (Jackson et al. 2012).

The criteria and approach we propose, for a paradigm shift towards *SIA*, integrates the dual and interdependent goals of using sustainable practices to meet rising human needs while contributing to resilience and sustainability across scales. Both of these, in turn, are required to sustain the future viability of agriculture. This paradigm shift aims at repositioning world agriculture from its current role as the world’s single largest driver of global environmental change, to becoming a critical agent of a world transition to global sustainability within the biophysical safe operating space on Earth.

A transformation to sustainable intensification is thus justified both by necessity (to safeguard global sustainability, a precondition for long-term agricultural viability) and by opportunity (to use sustainable practices as a vehicle for a second green revolution).

## Background

### The necessity of a transformation of sustainable intensification of agriculture

The case for intensification has been well articulated in the literature, both from a perspective of increased production, through high-yielding crops, increased irrigation, mechanization, and the role of chemicals that increase production levels (World Bank 2007), and from a conservation perspective, in terms of the millions of hectares of forests which otherwise would be converted into farm land, unquantifiable amount of ecosystem services saved, and of some 590 billion tons of CO_2_ prevented from being released into the atmosphere (Burney et al. 2010). We however underline the fact that much of such intensification has taken place with production increases being the primary, if not the sole, objective, whose negative consequences were understood after-the-fact and are now well documented.

Convincing evidence has emerged that humanity has entered the Anthropocene, where human pressures have reached a planetary scale in terms of ecosystem and resource constraints and rising risks of environmental shocks and large-scale tipping points (Lenton et al. 2008; Rockström et al. 2014; Steffen et al. 2015a; Waters et al. 2016). A rapid world transformation to global sustainability is increasingly acknowledged as necessary to enable human development within a functioning and healthy environment.

Agriculture is a primary driver of global change and is the single largest contributor to the rising environmental risks of the Anthropocene (Foley et al. 2011; Steffen et al. 2011; Struik et al. 2014). It is also in the Anthropocene that the challenge of feeding humanity needs to be resolved. The number of hungry people in the world remains at approximately 900 million (FAO, Ifad and WFP. 2013). At the same time, with rising living standards of the growing middle class, diets are shifting towards more livestock products that require more land and water resources than vegetarian sources of nutrition. In order to feed the world in 2050, global food production may have to increase by 60–110 % (Pretty 2008; IAASTD 2008; Tilman et al. 2011; Ray et al. 2013; Pardey et al. 2014). The challenge is further complicated by the need not only to produce more, but also to manage the entire food supply chain much more efficiently, reducing waste which has reached unacceptable proportions (estimated at 30%) along with promoting better distribution, access, and nutrition (FAO 2011a). This requires nothing less than a planetary food revolution which, for the foreseeable future, will largely be driven by the 2.5 billion smallholders that control 500 million small farms and which provide up to 80 % of the food supply in Asia and sub-Saharan Africa (FAO 2012) while residing in some of the world’s most social–ecologically vulnerable regions.

Today, approximately 40 % of the world’s terrestrial surface has been transformed to agriculture (crop, fiber, biofuel, and livestock production systems) (Ramankutty et al. 2008). Appropriate land for food production, however, is a finite resource and hence further expansion could compromise development within Earth’s safe operating space (approximately 25% of anthropogenic emissions of greenhouse gases are sequestered on land, of which all occurs in terrestrial non-cultivated ecosystems). If business-as-usual prevails, the expected range of cropland expansion (123–495 Mha per annum) would overshoot the preliminary estimate of the “safe operating space” of 1640 Mha well before 2050 (UNEP 2014).

Sustainable intensification of agriculture, in our proposed paradigm, aims at hunger reduction through biodiversity conservation that secures ecological functions in agricultural landscapes. It will require well-informed regional and targeted solutions (Tscharntke et al. 2012) drawing upon the strengths of both land-sparing and land-sharing approaches underpinned by strategic land-use planning and allocation (Law et al. 2015) across local, regional, and basin scales. Fischer et al. (2008) conclude that land sparing is readily compatible with optimization methods that attempt to allocate land uses in the most efficient way, while sustainable agro-ecological systems emphasize heterogeneity, resilience, and ecological interactions between farmed and unfarmed areas. Both social and biophysical factors influence which approach is feasible or appropriate in a given landscape. Our approach in this paper seeks to draw upon the strengths of each approach, although the focus of this paper is on transforming agricultural systems into sustainable agro-ecological systems. As mentioned above, however, conservation measures including protected area habitats, areas co-managed with local communities, and indigenous reserves are all potentially viable sustainable intensification strategies (Phalan et al. 2011; Garnett et al. 2013).

Our current agricultural inputs are also a challenge. Agriculture is the single largest user of freshwater in the world, with 70 % of the totally withdrawn water of almost 6000 km^3^ year^−1^ being diverted for agriculture (Kabat 2013), which has resulted in approximately 25 % of the world’s major river basins no longer reaching the ocean (Comprehensive Assessment of Water Management in Agriculture 2007). Agriculture is the world’s largest contributor to altering the global nitrogen and phosphorus cycles (Carpenter 2005). Anthropogenic uptake of N from the atmosphere (for industrial and intentional biological fixation of N) today exceeds the natural global uptake of N for biomass growth (Galloway and Cowling 2002; Gruber and Galloway 2008) and currently at approximately 150 Tg N year^−1^ the global uptake far exceeds the boundary value of 62–82 Tg N year^−1^ (Steffen et al. 2015a).

Although the focus of this paper is on sustainability as the strategy for productive agriculture, it is recognized that a case for sustainable intensification must also tackle the challenge of improving the health and livelihoods of the 2.5 billion smallholder farmers who are the primary stewards of our natural resources. As highlighted in the Global Nutrition Report (IFPRI 2015), improving nutrition status reduces disease burdens, increases income, improves life expectancies, and provides a host of additional socioeconomic benefits to families and communities. These benefits are essential drivers of sustainable development. A key strategy is investing in food that is healthy for people and planet, where nutritional food, low in refined sugars, fats, and meat, can help combat malnourishment and obesity and reduce emissions of greenhouse gases and resource footprints (Tilman and Clark 2014; IFPRI 2015).

Together, these social–ecological pressures pose an unprecedented challenge for the global food system, and we can see no other pathway to resolve it other than adopting a paradigm of sustainable intensification, with a dual purpose of (i) enabling a step-change in productivity and resilience and (ii) averting unacceptable global environmental risks.

### The way forward: transforming sustainable intensification of agriculture

Recent efforts in defining *SIA* (Royal Society 2009; Conway et al. 2010; Godfray et al. 2010; Rockström and Karlberg 2010; Tilman et al. 2011; Pretty et al. 2011; Garnett et al. 2013; Godfray and Garnett 2014) provide an emerging framework built around the simple principle whereby ‘yields are increased without adverse environmental impact and without the cultivation of more land’ (Royal Society 2009). Our conclusion is that these definitions are either not concrete enough or only partial. World agriculture must now meet social needs and fulfill sustainability criteria that enables food and all other agricultural ecosystem services (i.e., climate stabilization, flood control, support of mental health, nutrition, etc.) to be generated within a safe operating space of a stable and resilient Earth system, which in turn can be defined from Earth system science applying the planetary boundary framework (Table 1). This is a comprehensive definition of sustainable intensification of agriculture in the Anthropocene.

**Table 1:** The implications of the planetary boundary process for *SIA*

Planetary boundary process		Proposed boundary level (range of uncertainty)	Current level	Politically agreed/proposed boundary	Implication for *SIA*
Climate change		350 ppm CO_2_ (350–450 ppm) <1 W m^2^ (<1–1.5 W m^−2^)	396.5 ppm	Keep global average temperature rise <2 °C compared to pre-industrial levels Corresponding to ~400 ppm CO_2_ Translates to 1000 Gt CO_2_ remaining global carbon budget from 2011 onwards	50–80% reduction of CO_2_ emissions from energy use by 2050 (compared to 1990); Transform agriculture from world’s single largest carbon source to major carbon sink in soils
Land-use change	Global	Maintain >75 % forest cover for critical Earth system regulating forest systems	62 %	REDD+	Drastically reduced, and in most regions zero expansion of agricultural land
	Regional	Maintain 85 % rainforest, 85 % temperate forest: 50 % boreal forest		Aichi targets (17 % land ecosystems set aside as protected areas) Proposed SDG goals on halting biodiversity loss	
Global freshwater use	Global	Maximum 4000 km^3^ year^1^ of consumptive use	2600 km^3^ year^1^		50 % increase in water productivity by 2030
	Regional	Secure minimum volumes of environmental water flows in rivers 25–55 % maximum withdrawals of blue water in rivers (25–85 %)		River basin plans	Limit to runoff withdrawals in rivers
Biosphere integrity	Global	Genetic diversity: keep extinction rate <10 E/MSY	100–1000 E/MSY	Aichi targets Proposed SDG goal No 15 aimed at halting biodiversity loss	Zero loss of biodiversity in agricultural landscapes Adopt watershed and catchment management practices that build ecological landscape resilience
	Regional	Functional diversity: Biosphere integrity index >90 (90–30 %)		Aichi targets	
Interference N/P cycles	P Global *Oceans*	P flows from land to oceans <11 Tg P year^1^ (11–100 Tg P year^1^)	~22 Tg P year^1^		Close nutrient loops; not increase overall P use, Raise N and P use per ha in developing countries; reduce in developed countries
	P Regional *freshwater*	P flows from fertilizers to erodible soils <3.72 Tg year^1^ (3.72–4.84 Tg year^1^)—Global average but regional distribution is critical for impacts	~14 Tg P year^1^		
	N Global	Industrial and agricultural biological fixation of N < 44 Tg N year^1^ (44–62 Tg N year^1^)	~150 Tg N year^1^		
Novel entities		Reduce loading of novel, anthropogenic chemical compounds in the biosphere			Minimze leakage of agricultural chemicals

Recognizing the central role agriculture plays in determining and regulating Earth’s resilience, and the sustainability criteria for agriculture (outlined in Table 1), there is a strong case for adopting sustainable intensification of agriculture as the strategy to meet twin objectives for people and the planet. The “human goal,” adopted by the UN Sustainable Development Goals (SDGs) in 2015, is to eradicate hunger and poverty by 2030 (which will require >50 % increase in food production). The global sustainability goal (as defined by Table 1) is supported by the SDG goals and targets 2-*Healthy food for all*, 6-*Sustainable freshwater*, 12-*Sustainable Consumption and Production*, 13-*Decarbonising climate system under* 1.5–2 °C, 14-*Sustainable oceans*, and 15-*Halt biodiversity loss*) and can only be translated as the UN SDGs setting out to feed humanity this within a safe operating space of a stable and resilient Earth system. Together, these integrated goals will require a doubly green revolution (Conway 1997) within ambitious and absolute targets for sustainability: in principle (1) net zero emissions of greenhouse gases, (2) very low or zero expansion of agriculture into remaining natural ecosystems, while restoring others providing vital ecosystem services, (3) zero loss of biodiversity, (4) drastic reduction in excessive use of N and P (recycling nutrient flows), and (5) major improvement in water productivity and safeguarding of environmental water flows. These will require, among others, conducive legal and institutional frameworks, incentives, rights, infrastructure, and support services that farmers will need for implementation.

From these social–ecological criteria emerges a clear definition of sustainable intensification: adopting practices along the entire value chain of the global food system that meet rising needs for nutritious and healthy food through practices that build social–ecological resilience and enhance natural capital within the safe operating space of the Earth system.

#### Nature-based solutions for sustainable intensification of agriculture to build prosperity and resilience

Evidence increasingly shows that sustainable agricultural practices can raise productivity and meet sustainability criteria (Pretty 2008; Royal Society 2009; Conway et al. 2010; Godfray et al. 2010; Rockström and Karlberg 2010; Tilman et al. 2011; Pretty et al. 2011; Garnett et al. 2013; Ponisio et al. 2015). A recent WRI report (2013) documents a worldwide range of sustainable management practices of land, water, and biodiversity in agro-ecosystems that increase productivity. A key part of the journey to long-term *SIA* requires safeguarding not only local (on-farm) productivity through sustainable practices, but also ecological functions across scales, from watershed, to basin, region, and Earth system scales, to avoid, e.g., loss of rainfall during future growing seasons. It furthermore requires building the capacity to deal with rising frequency and amplitude of shocks and stresses as a result of global changes (e.g., droughts and floods exacerbated by climate change; disease outbreaks promoted by globalization).

For example, with rising risks of water shocks at the local scale—droughts, floods, and dry spells, it is increasingly important to manage water across scales—from local farm fields to watersheds and river basins. Spatial planning strategies are required to safeguard multi-functional landscapes, with a diverse set of ecosystems that are able to dampen the effects of storm-floods and maximize sub-surface flows of water rather than erosive surface runoff. Wetlands, meandering rivers, forests, and landscape mosaics are important natural capital assets that build resilience. Moreover, watershed and river basin management is required to safeguard rainfall. In many parts of the world, a large portion of rainfall (often >50 %) is convective, originating from local scale to meso-scale vapor flows, in particular from upwind evaporating forests contributing moisture flows that generate rainfall downwind. This so-called moisture feedback is common to the Sahel region where moisture from the West African rainforests in the south provides rainfall on the semi-arid savannah in the north. These examples demonstrate the importance of managing water at the watershed and regional scale in order to secure rainfall and therefore future food production at the local scale. This landscape approach needs to be nurtured and facilitated by a social–ecological framework for policy design and on-ground implementation (IAASTD 2008; Garnett et al. 2013; Godfray and Garnett 2014) (see Box 1).

**Box 1:** China’s dream: ecosystem function conservation areas

China is experiencing some of the world’s most extreme challenges of environment and human development. There is now open recognition, at the highest levels of government, that environmental security is vital to national security and economic prosperity (Daily et al. 2013). In the spring of 2013, the National Development and Reform Commission declared China’s Dream: “to become the Ecological Civilization of the 21st Century.” The backdrop to China’s Dream is that ecologically vulnerable areas account for more than 60 % of the country and cannot sustain current human impacts. Agricultural security—and ecological security more generally—is at high risk, with severe biodiversity loss, soil erosion, flooding, sandstorms, and water and air pollution. With the world’s largest population (over 1.35 billion), the second largest land area, and the second largest economy, the stakes are high.
In support of China’s Dream, leaders are fostering intense policy innovation, pioneering new mechanisms for achieving the twin goals of securing the environment and human wellbeing. What is learned in China will have relevance everywhere.
Ecosystem Function Conservation Areas (EFCAs) are a new system of zoning land so as to focus conservation and restoration in places with highest return-on-investment for public benefit, to halt and reverse degradation of vital ecosystems and their life-support services, especially to poor and vulnerable people (NRDC 2013; CCICED 2014). The zoning is also meant to help secure people from flooding, improve drinking and irrigation water supply, maintain efficient hydropower production, protect biodiversity, stabilize climate, reduce sand storms and soil loss, and create more sustainable agricultural systems (see below figure).
Figure showing China’s new Ecosystem Function Conservation Areas (EFCAs), zoned to protect nationally critical biodiversity and ecosystem services, and to alleviate poverty, now span 49 % of the country. The Natural Capital Project’s InVEST models were co-developed with the Chinese Academy of Sciences and are used to define the locations of EFCAs. China has invested over US$150 billion in restoring natural capital since 2000, through a suite of pioneering initiatives. Now entering a new phase of investment, over 200 million people are being paid to perform restoration and conservation activities. Figure courtesy of H. Zheng and Z. Ouyang, Research Center for Eco-Environmental Sciences, Chinese Academy of Sciences (MEP & CAS 1998; State Council of China 2010).
EFCAs are also a way of focusing poverty alleviation efforts in places where the stakes are highest, both for local residents and for distant beneficiaries of ecosystem services. EFCAs encompass rural areas in deep poverty that face great challenges in harmonizing people and nature. The government aims to change the economic structure of these regions to increase local household income while making local households’ rural livelihoods more sustainable.
Implementing EFCAs involves new, experimental compensation mechanisms, whereby regional beneficiaries—for example, Beijing—invest in the transformation to more sustainable livelihoods and improvements in wellbeing among the landholders producing the ecosystem services. EFCAs are expanding in both biophysical and financial terms. They spanned 27 % of the country in 2008, 40 % in 2010, and grew to 49 % of the country in 2015. Financial transfer payments increased from 6 billion Yuan RMB (to 221 counties) in 2008 to 48 billion Yuan RMB (to 512 counties) in 2014, for a total of 200 billion Yuan RMB (USD 32 billion) since inception.
While EFCA initiatives are driving massive scientific and policy shifts, there is still little understanding of their local costs of implementation, their success in reversing environmental degradation, or their effects on poor and vulnerable populations. The initiatives represent a new paradigm for integrating conservation and human development, in China and potentially elsewhere. Success hinges on careful testing, evaluation, and refinement.
Further, while the idea of a green GDP has been discussed for decades, China is the first nation to implement it. In March 2014, the Ministry of Environmental Protection of China approved reporting Gross Ecosystem Product (GEP) alongside Gross Domestic Product at all levels of government, from local to national. GEP is the total value of final ecosystem goods and services to human welfare, including production of agricultural goods, as well as generation of regulating services and cultural values (Ouyang et al. 2013). The objective of GEP accounting is to determine the total economic value of ecosystem contributions to human wellbeing, to build the links between ecosystem service providers and beneficiaries, and to assess the achievements of ecological protection and government management, instead of only GDP.

*SIA* requires a radical refocusing of food production that encapsulates the twofold aims of increasing yields and the ecosystem services provided by agriculture (Godfray and Garnett 2014). In some areas, increases in yield will be compatible with environmental improvements. In others, yield reductions or land reallocation will be needed to ensure sustainability and deliver benefits such as biodiversity conservation, carbon storage, flood protection, and recreation (see Box 1). An overall increase in production does not mean that yields should increase everywhere or at any cost: the challenge is context and location specific. Hence, *SIA* is about strategic land-use planning to maintain and improve the interacting stocks and flows involving water, nutrients, energy, carbon, and biodiversity across landscape mosaics of natural, semi-natural, and agricultural land uses, so that multi-functionality of the whole landscape is manageable across scales from local to basin to national levels.

From a production perspective (see Box 2), *SIA* should now entail a three-step approach: (1) at the basis be as resource efficient as possible combining locally relevant crop and animal genetic improvement and practices that minimize inputs and close nutrient, carbon, and water cycles, (2) adopt practices that build landscape-scale resilience by sustaining ecosystem functions and services, such as water flows and biodiversity, and (3) connect thinking, planning, and practice across scales to fully grasp field to biome and global interactions in the Anthropocene. This must go with improved and more equitable access to knowledge and resources including land tenure, common property, markets, and social relations. Building on the work of Pretty et al. (2011) and others (e.g., van Noordwijk and Brussaard 2014; Tittonell 2014), a paradigm shift towards *SIA* translates to some key operational strategies:

**Box 2:** Smart solar pumps: a potential solution to groundwater exploitation in India

In Karnataka, southwest India, the local electric company is required to buy back surplus solar power from farmers—similar to programs in parts of Germany, Japan, and the United States. The buyback policy, signed by Karnataka’s governor in September 2014, is consistent with recommendations to treat solar power as a ‘cash crop.’ The rationale is that if farmers can make money by selling excess power, they then will have an economic incentive to irrigate their crops efficiently, thus helping to conserve groundwater and energy use.
Despite inheriting the world’s largest canal irrigation network built during British colonial rule, India has become the biggest groundwater irrigation economy, with nearly 20 million electric and diesel pumps irrigating more than 67 million hectares of land a year. Heavily subsidized pumps have driven groundwater depletion in western India and other parts of the country. An unreliable electric grid, bankrupted utilities, and power theft have contributed to the problem.
India’s National Solar Mission, which aspires to develop 22 gigawatts of solar power by 2020, largely by constructing massive solar power plants. However, India could achieve its solar goal with 2 million solar irrigation pumps instead and “put cash in farmers’ hands” in the process. The approach that is being promoted in Karnataka is presented. This approach of selling excess electricity back into the national grid could be used elsewhere in developing and emerging economies to drive significant decreases in CO_2_ emissions from fossil fuels used to pump groundwater, a shift to more sustainable utilization of groundwater, as well as enhanced food security.
Figure addressing the challenge of over-exploited groundwater reserves in India through the co-generation of power from solar panels for pump sets to pump water for irrigation and satisfy national energy requirements in India

- Plan and implement farm-level practices in the context of cross-scale interactions with catchments, biomes, and the landscape as a whole. Maximize farm-level productivity by maximizing ecological functions, from moisture feedback to disease abatement, across scales.
- Integrate ecosystem-based strategies with practical farm practices, where natural capital (soil, biodiversity, nutrients, water) and multi-functional ecosystems are used as tools to develop productive and resilient farming systems.
- Develop system-based farming practices that integrate land, water, nutrient, livestock, and crop management.
- Utilize crop varieties and livestock breeds with a high ratio of productivity to use of externally and internally derived inputs.
- Adopt circular approaches to managing natural resources (e.g., nutrient recycling) and mixing organic and inorganic sources of nutrients.
- Harness agro-ecological processes such as nutrient cycling, biological nitrogen fixation, allelopathy, predation, and parasitism.
- Assist farmers in overcoming immediate *SIA* adoption barriers and build incentives for their sustained adoption, rendering the ecological approach profitable in the long run (See Box 2).
- Build robust institutions of small farmers, led especially by women, which enable an equitable interface with both markets and government.

#### Sustainable intensification can deliver more food, better ecosystems, and improved livelihoods

Scientific and practical evidence clearly indicates that agriculture can shift from “foe,” in terms of being the single large contributing sector to global environmental risks, to “friend,” thereby contributing to global sustainability, and, in so doing, build natural capital and resilience, while increasing productivity and improving livelihoods (Pretty et al. 2006, Pretty 2008; IAASTD 2008; Foley et al. 2011; Pretty et al. 2011). The sources of sustainable practices range across all areas of agricultural development, in soil tillage systems, water resource management, crop and nutrient management, livestock practices, integrated landscape management, pest management, and management of ecosystem services are already evident and what is required is a scaling up. For example:

- The Comprehensive Assessment of Water Management in Agriculture (2007) showed that there is a large untapped potential in upgrading rainfed agriculture in savannah regions (covering 40 % of the Earth’s surface) by enhancing rainwater harvesting. As an example, in semi-arid areas of Niger and Burkina Faso, small-scale farmers use planting pits to harvest rain water and rehabilitate degraded land for the cultivation of millet and sorghum. In Burkina Faso alone, these practices have helped rehabilitate up to 300 000 hectares of land and produce an additional 80 000 tons of food per year (Reij et al. 2009). In addition, in southern Niger, farmers are innovatively regenerating and multiplying valuable trees on their lands, and this has improved about 5 million hectares while producing more than 500 000 additional tons of food per year resulting in improved food security for about 3 million people. Other ecosystem benefits registered included reduced wind speed and evaporation (Reij et al. 2009), and incomes for women from different products of baobab up to $210 per household per year.
- In Ethiopia, farmers capture flood water and runoff from ephemeral rivers, roadsides, and hillsides using temporary stone and earth embankments, to irrigate crops and pasture. In the central and western part of the country, total irrigated land is approximately 65 500 ha, and some 344 000 (approximately 90 %) of the households have benefited from doubling of sorghum yields as well as 75 % sustainable expansion production of pepper, onions, and tomatoes (Binyam and Desale 2015). Other ecosystem benefits have included improved moisture and fertility in the cultivated fields and reduction of downstream flooding (Awulachew 2010; Liniger et al. 2011).
- In Brazil, conservation agriculture (CA) which is practiced on over 25 million ha (accounting for over 25.5 % of arable land) is defeating erosion and drought. For example, severe drought in 2008–2009 caused an average yield loss of 50 % among conventional maize producers; producers who applied CA, however, experienced smaller losses of around 20 %, demonstrating greater resilience of the latter system (Altieri et al. 2012).
- Too often, agro-ecosystems have been considered as separate from other natural ecosystems and insufficient attention has been paid to the way in which services can flow to and from the agro-ecosystem to surrounding ecosystems. Recent research (Poppy et al. 2014) illustrates that an ecosystem services approach to food security using a case study from the Zomba district of Malawi allows key issues in food security/environmental stability to be addressed, including scale, the identity of beneficiaries, trade-offs, and the winners and losers from management and mitigation strategies. The study illustrates the power of an ecosystem services approach to strategic land-use planning and implementation.
- Science and innovation that strengthens sustainability, while improving productivity and on-farm profits, is possible. Such systems have been developed in Australia (Williams and McKenzie 2008a, b) and elsewhere and have been adopted by grain growers who are moving increasingly to conservation farming techniques, such as no-till farming—improved agronomy through more sophisticated crop rotations to minimize nutrient leakage and maximize nutrient cycling, interfaced with integrated weed and pest management options that rely less on chemicals.
- In the southern Indian state of Andhra Pradesh, a million farmers have come together, in an FAO-supported project, to restore depleted groundwater tables, adopting an approach to governing the commons delineated by Nobel Laureate Elinor Ostrom (World Bank 2010). Food security is increased, utilizing ecosystem services, without exhausting the endangered resource.
- Rehabilitating degraded landscapes in the Highlands is a high-priority of the Ethiopian government and its partners. Research by CGIAR Centers and programs working with national partners has helped lay the groundwork. An ICRISAT-led activity is promoting integrated watershed management in the Yewol watershed in the Amhara Regional State, Ethiopia. By strengthening local capacity, facilitating collective action, using research to identify niches for integration of technologies at farm and landscape scales, and introducing system compatible technologies, the project has led to improved productivity, crop diversification, improved downstream water availability, and strengthened livelihoods for an estimated 15 000 beneficiaries (Evaluation of WLE 2016, p. 52).

## Conclusions

### Challenges for science

Adopting a livelihood-centered paradigm for sustainable intensification within planetary boundaries is a major challenge for research and development that will require new approaches to how research for development is formulated, managed, and executed.

Pursuing *SIA* will entail approaches that integrate social and natural sciences, in solution-oriented knowledge generation that couples academic and practical knowledge through co-design and co-development of research. The implementation of *SIA* will require an understanding of the political economy in which food is traded and prices are determined and the business economy along the value chain from field to consumer. A major reason why farmers persist in growing water-intensive crops even in water-scarce regions is that State support for prices and procurement is limited to such crops and is not available for more ecologically appropriate crops such as pulses and millets. The result has been the emergence, for example of the “Punjab Water Syndrome,” where falling water tables combine dangerously with waterlogging in other parts of the state in India (Kulkarni and Shah 2013). However, the aforementioned could be addressed through innovative incentive-based approaches that result in distinct behavior changes (see Box 2).

A lasting paradigm shift will require the ability to place research into policy and enable large-scale change. Influencing policy requires an understanding of the power dynamics and political systems that both enable and undermine the shift to *SIA*, associated improvements in livelihoods, and protection of the environment. Institutional trust will need to be built among the many stakeholders in the food system, all of whom will be required to make compromises. While *SIA* needs to be central to the way we produce food in the future, it also needs to be integrated within a nexus of strategies aimed at achieving food system sustainability, in the broadest sense of the phrase (Godfray and Garnett 2014).

### Grand experiments for transformation, co-design, and learning

It should be recognized that *SIA* is a new, evolving concept, and its meaning and objectives subject to debate and contest. Sustainable intensification is only part of what is needed to improve food system viability and sustainability and is not synonymous with food security. Both sustainability and food security have multiple social, ethical, and environmental dimensions. Achieving a sustainable, health-enhancing food system for all will require more than just changes in agricultural production, essential though these are. Equally radical agendas will need to be pursued to reduce resource-intensive consumption and waste and to improve governance, e.g., on trade, incentives and equity. Much hope has been generated by India’s 12th Five Year Plan, which adopts a paradigm shift in water resource management, exactly along the lines proposed in this paper (Shah 2013). A promising development is the emphasis in the strategic plan of the CGIAR until 2030 (CGIAR 2015), which places reduced poverty, improved food and nutrition security for health, and improved natural resources and ecosystem services, as its three highest level system outcomes. As the CGIAR played a pivotal role in the 1st Green Revolution, this creates the potential framework for a 2nd Green Revolution based on *SIA* principles.

Similarly, FAO pursues a strategic transformation, which endorses an ecosystem approach in agricultural management for sustainable crop production intensification, provides associated policy advice, and envisages a vision of sustainable food and agriculture that merges access by all to nutritious food with ecosystem-focused natural resources management (FAO 2011b, 2014).

The shift outlined in this article demands a new framework for research and development. Major productivity enhancements are required, and the strategy is through sustainable intensification of agricultural practices for livelihoods that build farm, community, and biosphere resilience. New research and development is required to advance fresh integrated whole-of-systems approaches for sustainable intensification, which can inspire and influence all domains involved in agricultural development, from economics to biotechnology.

We believe one strategy forward is the investment in spatially concentrated major “grand experiments” where knowledge from different domains, ranging from irrigated to rainfed agriculture, ecology and agronomy, equity to business development, work together to pilot sustainable intensification at scale (e.g., in a region or basin), to pool experience, explore synergies and trade-offs, testing the hypothesis that sustainable intensification can deliver food, livelihoods, and resilience, while contributing to development within Earth’s safe operating space. These would be large R&D investments. They would deviate from the normal business-as-usual approaches of discipline by discipline, sector-by-sector, scale-by-scale approaches to agricultural development. They would be system-integrating and innovative ventures, and thus challenging but, as argued in this paper, necessary. Evidence strongly suggests that sustainable transformations of agricultural systems are direly and urgently required to meet World and Earth needs.
